# Reply to Cosio et al. Glycation of Nail Proteins as a Risk Factor for Onychomycosis. Comment on “Gupta et al. Diabetic Foot and Fungal Infections: Etiology and Management from a Dermatologic Perspective. *J. Fungi* 2024, *10*, 577”

**DOI:** 10.3390/jof11010047

**Published:** 2025-01-08

**Authors:** Aditya K. Gupta, Avner Shemer, Vasiliki Economopoulos, Mesbah Talukder

**Affiliations:** 1 Division of Dermatology, Department of Medicine, Temerty Faculty of Medicine, University of Toronto, Toronto, ON M5S 3H2, Canada; 2 Mediprobe Research Inc., London, ON N5X 2P1, Canada; 3 Department of Dermatology, Sheba Medical Center, Tel-Hashomer, Ramat-Gan 52621, Israel; ashemer1@gmail.com; 4 Sackler Faculty of Medicine, Tel Aviv University, Tel Aviv 6997801, Israel; 5 Department of Medical Biophysics, Schulich School of Medicine and Dentistry, Western University, London, ON N6A 3K7, Canada; 6 School of Pharmacy, BRAC University, Dhaka 1212, Bangladesh

We find the comment on the article titled “Diabetic Foot and Fungal Infections: Etiology and Management from a Dermatologic Perspective” informative and wish to thank the authors for their added insight on this complex topic [1,2]. We agree that advanced glycation products formed within the diabetic patient may play a role directly in the development of onychomycosis. Understandably, there are multiple mechanisms at play for the increased risk of infection and complications in this patient population. The immunological pathways described are only one part of a much larger picture. Further knowledge on the interplay of these pathways, as well as any additional mechanisms, will be of the utmost importance to reducing the burden of onychomycosis in diabetic patients.
